# The Legislative Trajectory of Korea’s Nursing Act: Insights From the Multiple Streams Framework

**DOI:** 10.1155/jonm/7849085

**Published:** 2026-06-08

**Authors:** Donguk Lee

**Affiliations:** ^1^ Department of Public Administration, Kyung Hee University, Seoul, South Korea, khu.ac.kr

**Keywords:** healthcare governance, multiple streams framework, nursing act, policy entrepreneur, policy process, presidential systems, stream convergence

## Abstract

**Background:**

The enactment of the Korean Nursing Act emerged through a prolonged and conflict‐laden policy process shaped by workforce instability, interprofessional conflict, and changing political conditions within the Korean healthcare system. Recent healthcare crises, including the COVID‐19 pandemic and the 2024 healthcare service vacuum, further intensified policy attention toward nursing workforce governance and healthcare system sustainability.

**Aim:**

This study examines the legislative trajectory and postenactment policy developments of the Korean Nursing Act between 2005 and 2025 through the analytical lens of Kingdon’s Multiple Streams Framework (MSF).

**Method:**

Using qualitative document analysis, this study analyzed legislative bills, National Assembly records, government documents, organizational statements, media reports, and academic literature related to the Nursing Act. The analysis focused on interactions among the problem, policy, and politics streams, as well as the role of policy windows and policy entrepreneurs throughout the legislative process.

**Results:**

The findings indicate that longstanding issues—including poor working conditions, ambiguity in nursing roles, workforce instability, and concerns regarding healthcare sustainability—gradually evolved from profession‐specific grievances into broader public policy concerns. Focusing events such as the COVID‐19 pandemic and the 2024 healthcare service vacuum substantially strengthened the problem stream and altered the political environment surrounding the legislation. The policy stream evolved through repeated revision, negotiation, and conflict over issues including professional boundaries, the “community” clause, and physician assistant (PA) nurses. Within the politics stream, partisan realignment, organized interest‐group conflict, and executive intervention significantly influenced the trajectory of policy change. Although convergence among the problem, policy, and legislative political streams enabled passage of the Nursing Act during the 21st National Assembly, presidential veto power ultimately disrupted policy adoption. Enactment during the 22nd National Assembly became possible only after crisis‐driven political realignment weakened institutional resistance and enabled renewed stream coupling.

**Conclusion:**

This study demonstrates that stream convergence within healthcare policymaking processes may remain unstable under presidential systems characterized by strong executive authority. The findings extend existing applications of the MSF by illustrating how executive veto power may destabilize policy convergence even after substantial legislative agreement has emerged.

**Implications for Nursing Management:**

The study provides important implications for healthcare workforce governance, nursing leadership, and policy advocacy in aging societies facing healthcare workforce shortages. The findings suggest that effective nursing policy reform requires sustained interprofessional coordination, strategic policy entrepreneurship, and governance systems capable of responding flexibly to healthcare crises and workforce instability.

**Highlights**
• Examines the Korean Nursing Act enactment via the Multiple Streams Framework.
• Identifies key social, policy, and political factors driving legislative success.
• Highlights the role of policy entrepreneurs and external crises in stream alignment.
• Provides insights for global health policy in aging societies with workforce issues.

## 1. Introduction

### 1.1. Background and Rationale

The enactment of the Nursing Act in South Korea represents the culmination of a prolonged and conflict‐laden policy process shaped by competing professional interests, institutional constraints, and shifting political conditions. Although demands for an independent legal framework for nursing emerged within the nursing profession as early as the 1970s, substantive legislative debate accelerated only after the mid‐2000s [1–5]. Since the first Nursing Act bill was introduced during the 17th National Assembly in 2005, successive versions of the legislation were repeatedly proposed, revised, delayed, or rejected across multiple legislative sessions [6–9]. These repeated legislative failures reflected not only procedural obstacles but also deeper structural tensions embedded within the Korean healthcare system [10–13].

At the center of the debate were longstanding issues within the nursing profession, including poor working conditions, unclear professional boundaries, and chronic undervaluation of nursing labor [14–23]. Excessive workloads, high nurse‐to‐patient ratios, emotional exhaustion, and persistent turnover problems intensified concerns regarding workforce sustainability and patient safety [24–35]. At the same time, the legal definition of nurses under the Medical Service Act remained largely physician‐centered, positioning nurses primarily as assistants despite the increasing complexity and autonomy of contemporary nursing practice [36–42].

Demographic and institutional changes further intensified pressure for legislative reform. Rapid population aging, increasing prevalence of chronic diseases, and expansion of community‐based healthcare exposed limitations within Korea’s existing healthcare governance structure [22, 43–45]. Supporters of the Nursing Act argued that these changes required a clearer legal and institutional foundation for nursing roles and responsibilities. However, legislative efforts encountered strong resistance from physician organizations, particularly the Korean Medical Association (KMA), which argued that an independent Nursing Act would destabilize the healthcare system and intensify interprofessional conflict [46–58]. Political tensions surrounding the legislation reached their peak during the 21st National Assembly, when the Nursing Act passed the plenary session but was subsequently vetoed by the president [59–61].

A major turning point emerged in 2024 following the healthcare service vacuum triggered by the collective resignation of medical residents protesting the expansion of medical school quotas [62–66]. The crisis significantly increased the visibility of nurses’ roles in sustaining healthcare delivery and altered the political environment surrounding the legislation. In this context, the 22nd National Assembly ultimately passed the Nursing Act with bipartisan support, leading to the formal enactment of the legislation [67–71].

The legislative trajectory of the Nursing Act provides an important case for examining how policy change occurs under conditions of institutional conflict, uncertainty, and shifting political opportunity. This study analyzes the enactment process using Kingdon’s MSF, which conceptualizes policy change as the result of interactions among the problem, policy, and politics streams [72]. The MSF is particularly useful for explaining why certain policy proposals succeed only after prolonged periods of failure and how focusing events and political realignments may substantially alter policy trajectories.

Existing studies on the Nursing Act have focused primarily on specific legislative disputes, legal interpretation, or interprofessional conflict [4, 39, 46, 73–75]. Relatively limited attention has been directed toward explaining the broader policy process through which stream convergence, focusing events, political realignment, and executive intervention collectively shaped legislative outcomes across multiple National Assembly periods. In particular, few studies have examined how executive veto power within a presidential system may destabilize stream convergence even after substantial legislative alignment has been achieved.

Accordingly, this study traces the legislative trajectory of the Nursing Act across multiple legislative periods in order to examine how the three streams evolved independently, how they interacted over time, and how policy entrepreneurs contributed to stream coupling during critical policy windows. The study further explores how executive veto power and crisis‐driven political realignment influenced the trajectory of policy convergence within the Korean presidential system.

### 1.2. Research Objectives

This study aims to analyze the legislative process leading to the enactment of the Nursing Act in South Korea through the analytical lens of Kingdon’s MSF. Specifically, the study pursues the following objectives:

1. To examine how the problem, policy, and politics streams evolved throughout the legislative process;
2. To identify critical moments of stream convergence and analyze how policy windows emerged;
3. To investigate the role of policy entrepreneurs in facilitating stream coupling and advancing legislative change;
4. To explore broader implications of executive veto power and political realignment for policy processes within presidential systems.

By addressing these objectives, the study seeks not only to explain the enactment of the Nursing Act but also to contribute to broader discussions regarding healthcare governance, workforce policy conflict, and agenda‐setting processes in complex political environments.

## 2. Theoretical Background

### 2.1. Kingdon’s Multiple Streams Framework

Kingdon’s MSF explains policy change as the result of interactions among three relatively independent streams—the problem stream, the policy stream, and the politics stream—which converge during limited windows of opportunity known as policy windows [72]. The framework was originally developed to explain why certain policy issues gain governmental attention and why some policy proposals advance despite conditions of uncertainty, conflict, and institutional fragmentation [72].

The problem stream refers to the process through which particular social conditions come to be recognized as public policy problems requiring governmental intervention [72]. Within the MSF, problems are not viewed as purely objective conditions, but as issues that acquire political salience through interpretation, framing, and selective attention [76, 77]. Problem recognition is shaped through mechanisms such as indicators, focusing events, and policy feedback [72, 78]. Indicators reveal the severity or trajectory of a problem, focusing events generate heightened political and public attention, and policy feedback emerges when existing institutional arrangements expose limitations or unintended consequences [72, 78, 79].

The policy stream concerns the generation, refinement, and competition of policy alternatives within specialized policy communities composed of experts, bureaucrats, academics, and advocacy groups [72]. Kingdon described this process as a “primeval soup” in which ideas continuously evolve, combine, and compete for political acceptance. Policy alternatives are more likely to survive when they are perceived as technically feasible, politically acceptable, and compatible with prevailing institutional values [72, 80, 81].

The politics stream reflects broader political conditions, including national mood, organized political pressure, partisan dynamics, leadership change, and shifts within governing institutions [72, 82, 83]. Unlike the problem and policy streams, which are more strongly influenced by expertise and issue framing, the politics stream is shaped primarily by political incentives, electoral considerations, and power relations among organized actors.

Policy change becomes possible when the three streams converge during a policy window [72]. Policy windows may emerge through crises, political realignments, elections, institutional turnover, or other focusing events that alter political attention and policy feasibility [84–89]. However, policy windows are temporary and unstable, and convergence among streams does not necessarily guarantee policy adoption.

Policy entrepreneurs play a critical role in facilitating stream coupling [72, 83, 84]. These actors invest time, political resources, expertise, and organizational capacity to connect problems, policy alternatives, and political opportunities. Previous MSF scholarship has emphasized the importance of strategic framing, coalition‐building, and agenda‐setting in enabling successful stream convergence [90, 91].

Subsequent scholarship has refined and expanded the MSF in several respects. Zahariadis [83] emphasized the role of ambiguity and institutional fluidity within policymaking processes, particularly under conditions of high political conflict and uncertain decision‐making environments. Knaggård [92] further highlighted the strategic role of actors involved in defining and framing policy problems, drawing attention to how problem construction itself may shape agenda‐setting dynamics. Cairney and Jones [76] demonstrated the continued applicability of the MSF in explaining complex and nonlinear policy processes across diverse institutional contexts.

Previous studies have also demonstrated the applicability of the MSF across diverse institutional settings, including European Union policymaking processes [93]. The MSF is particularly useful for analyzing healthcare policy processes characterized by prolonged conflict, institutional fragmentation, and competing professional interests [81, 90, 94, 95]. In the case of the Nursing Act, disputes surrounding professional boundaries, healthcare governance, and the interpretation of key policy terms illustrate the importance of ambiguity within the policy process. In particular, controversy surrounding the term “community” demonstrates how competing interpretations of policy language may destabilize stream convergence and intensify political conflict [96, 97].

At the same time, the Korean case suggests that institutional characteristics associated with presidential systems may significantly influence stream stability during later stages of policymaking. Although stream convergence may occur within the legislative arena, strong executive veto power may interrupt or reverse policy adoption even after substantial political agreement has emerged. In this respect, the present study extends the application of the MSF by suggesting that executive veto authority may function as a destabilizing factor in stream coupling processes under presidential systems.

### 2.2. Previous Studies on the Enactment of the Nursing Act

Research concerning the Nursing Act in South Korea has primarily focused on the necessity of independent nursing legislation, interprofessional conflict, and legal or institutional implications associated with nursing workforce governance [73, 98–105]. Previous studies have emphasized structural problems within the nursing profession, including poor working conditions, ambiguity in professional roles, workforce instability, and concerns regarding patient safety [73, 98–103]. Other studies have examined the political and institutional conflicts surrounding the legislative process, particularly tensions between nursing organizations and physician groups [104, 105].

At the same time, a growing body of scholarship has applied Kingdon’s MSF to healthcare and social policy processes in Korea [106–112]. Previous studies have used the MSF to examine issues such as health insurance reform, infectious disease governance, mental health legislation, and healthcare privatization debates. These studies collectively demonstrate the usefulness of the MSF for analyzing policy change under conditions of institutional conflict, ambiguity, and shifting political opportunity.

However, existing research on the Nursing Act has several limitations. First, much of the literature has focused on normative debates regarding the necessity or legitimacy of the legislation rather than systematically examining the broader policy process through which enactment occurred. Second, previous analyses have tended to focus on specific legislative disputes or isolated political events rather than tracing long‐term interactions among the problem, policy, and politics streams across multiple legislative periods.

In addition, relatively little attention has been paid to how executive intervention, prolonged interprofessional conflict, and repeated failures of stream convergence shaped the legislative trajectory of the Nursing Act. Existing MSF studies within the Korean healthcare policy context have also paid limited attention to how institutional characteristics associated with presidential systems may affect stream stability and policy adoption during later stages of the policymaking process.

This study addresses these gaps by providing a longitudinal analysis of the Nursing Act’s legislative trajectory from the perspective of the MSF. In particular, the study examines how repeated failures of convergence, executive veto power, and crisis‐driven political realignment influenced the eventual enactment of the legislation. By doing so, the study seeks to contribute not only to the literature on nursing policy and healthcare governance but also to broader discussions regarding stream instability, policy entrepreneurship, and policy change within presidential systems.

## 3. Research Method

This study employed a qualitative case study approach to examine the legislative trajectory of the Nursing Act in South Korea through the analytical lens of Kingdon’s MSF. The MSF is particularly suitable for analyzing complex and conflict‐driven policy processes because it emphasizes ambiguity, temporal fluidity, and the nonlinear interaction among multiple streams within policymaking environments characterized by institutional fragmentation and stakeholder competition [72, 76, 113].

Data were collected through qualitative document analysis using publicly accessible materials related to the legislative trajectory and postenactment developments of the Nursing Act between 2005 and 2025. The analyzed materials included legislative bills and amendment proposals submitted to the National Assembly, plenary session records and committee meeting transcripts, official policy documents and press releases issued by relevant government agencies such as the Ministry of Health and Welfare, statements and publications released by stakeholder organizations including the Korean Nurses Association (KNA) and the KMA, national and healthcare‐related media reports, academic journal articles, policy reports, and related legal and institutional documents.

To improve analytical rigor and transparency, documents were selected according to three criteria: (1) direct relevance to the Nursing Act and its policy development trajectory, (2) substantive discussion of nursing‐related policy conflict, stream interaction, or legislative change, and (3) temporal relevance to major policy events associated with the enactment process. The study reviewed approximately 200 documents, including legislative records, government reports, stakeholder statements, media articles, policy reports, committee transcripts, and academic publications spanning approximately two decades of policy development, enabling a comprehensive examination of the evolving policy and governance process surrounding the Nursing Act.

The collected materials were analyzed using deductive thematic coding based on the core analytical categories of the MSF: Problem stream, policy stream, politics stream, policy window, and policy entrepreneurs. During the first stage of analysis, documents were categorized according to the stream to which they were most closely related. At the same time, interactions and overlaps among streams were also considered throughout the analytical process. In the second stage, recurrent themes, focusing events, actor strategies, and institutional responses were identified through iterative comparative analysis across sources. Particular attention was paid to moments in which interactions among streams intensified, converged, or shifted over time.

In analyzing the problem stream, the study focused on how issues such as poor working conditions, workforce shortages, ambiguity in nurses’ legal roles, and concerns regarding patient safety came to be recognized as public policy problems. Specific attention was given to major focusing events—including the MERS outbreak, the COVID‐19 pandemic, and the 2024 healthcare service vacuum—as well as statistical indicators and policy feedback that elevated the salience of nursing‐related issues.

The policy stream analysis examined how legislative alternatives evolved over time through proposal, revision, negotiation, and contestation. This included analyzing the development of competing versions of the Nursing Act, debates surrounding controversial provisions such as the term “community,” and policy discussions regarding PA nurses and clinical support duties.

The politics stream analysis focused on changes in public opinion, interprofessional conflict, party positioning, electoral dynamics, and executive‐legislative relations. Particular attention was given to the role of organized interest groups, partisan realignment, and presidential veto power in shaping the trajectory of the legislation.

The study also examined the opening and closure of policy windows by identifying critical moments in which the three streams converged or diverged. In this context, policy entrepreneurs were analyzed in terms of their agenda‐setting activities, coalition‐building efforts, framing strategies, and capacity to mobilize political and public support.

Media reports were used as supplementary sources to identify framing patterns, stakeholder positions, major conflict narratives, and temporal shifts in policy discourse surrounding the Nursing Act.

To strengthen the credibility of the analysis, triangulation was employed through cross‐verification of information derived from multiple types of sources. In addition, actor influence levels presented in Table 1 were assessed based on four criteria: (1) agenda‐setting capacity, (2) legislative influence, (3) public mobilization and coalition‐building ability, and (4) direct impact on policy outcomes. These criteria were used to comparatively evaluate the relative influence of major actors involved in the legislative process.

**TABLE 1:** Key actors and their roles in the legislative process of the Nursing Act.

Key actor	Position	Core claims/logic	Major activities/strategies	Level of influence
Korean Nurses Association	Supportive	‐ Improve nurses’ working conditions ‐ Clarify the scope of practice ‐ Promote public health ‐ Prepare for an aging society	‐ Supported bill proposals ‐ Public campaigns ‐ Demonstrations ‐ Political lobbying ‐ Legal actions and coalitions	Very High

Korean Medical Association	Opposed	‐ Fear of the collapse of the healthcare system ‐ Accusations of occupational egoism ‐ Concerns over illegal practice ‐ Claims the Medical Service Act is sufficient	‐ Public statements ‐ Protests ‐ Lobbying ‐ Emergency committees ‐ Strike threats	Very High

Korean Licensed Practical Nurses Association	Effectively opposed (conditionally supportive)	‐ Opposes academic restrictions ‐ Demands better treatment and recognition	‐ Expressed an independent stance ‐ Formed or opposed alliances with other organizations	Moderate

Ministry of Health and Welfare	Mediator/Conditional Acceptance	‐ Minimize occupational conflict ‐ Protect public health ‐ Ensure policy rationality	‐ Proposed compromise solutions ‐ Issued official opinions on bills ‐ Announced related policies	High

Democratic Party of Korea	Supportive (Leading)	‐ Improve nurses’ treatment ‐ Emphasize the need for healthcare reform	‐ Led bill proposals ‐ Adopted party platform ‐ Spearheaded passage in the National Assembly	Very High

People Power Party	Changing Position (Initial partial support ⟶ Opposition ⟶ Final agreement)	‐ Initially: co‐sponsored bill ‐ Middle: supported veto citing occupational conflict ‐ Later: negotiated revisions to resolve healthcare vacuum	‐ Led amendment negotiations ‐ Managed committee processes ‐ Facilitated compromise and passage	Very High

Office of the President	Changing Position (Veto ⟶ Acceptance)	‐ 21st Assembly: veto due to occupational conflict and concerns over noninstitutional settings ‐ 22nd Assembly: accepted compromise due to political shifts	‐ Announced veto ‐ Decided through Cabinet meetings ‐ Accepted bipartisan agreement	Very High

Pro‐Nursing Members of Parliament	Supportive	‐ Strengthen nursing professionalism ‐ Improve public health	‐ Sponsored legislation ‐ Hosted forums ‐ Persuaded lawmakers within the Assembly	High

National Coalition for the Enactment of the Nursing Act	Supportive	‐ Emphasized the national necessity of enacting the Nursing Act	‐ Coalition‐building ‐ Public campaigns ‐ Organized demonstrations	Moderate

Throughout the analysis, emphasis was placed on both the relative independence and the interaction of streams over time, as well as on the fragmented and nonlinear nature of policymaking processes. The analysis primarily focused on the legislative trajectory leading to the enactment of the Nursing Act, while selected postenactment developments emerging in 2025 were additionally incorporated to contextualize ongoing governance and implementation‐related conflicts.

## 4. Research Findings: An MSF‐Based Analysis of the Legislative Process of the Nursing Act

### 4.1. Problem Stream: Recognition and Emergence of Nursing Issues

The policy problems underlying the enactment of the Nursing Act emerged gradually over several decades and gained increasing political salience through the accumulation of institutional tensions, workforce instability, and a series of major focusing events [114–120]. These problems were closely associated with deteriorating working conditions for nurses, ambiguity in professional roles, concerns regarding patient safety, and broader structural changes within the healthcare system.

One of the most persistent issues involved poor working conditions within the nursing profession. Excessive workloads, high nurse‐to‐patient ratios, emotional exhaustion, and chronic turnover were repeatedly identified as structural weaknesses of the Korean healthcare system [14–35]. These conditions contributed not only to burnout and workforce instability but also to difficulties in retaining experienced clinical nurses. Nursing organizations, particularly the KNA, consistently framed these issues not merely as labor concerns but as systemic threats to healthcare quality, continuity of care, and long‐term sustainability [7].

Another major source of policy conflict involved ambiguity surrounding nurses’ legal roles and responsibilities. Under the existing Medical Service Act, nurses were primarily defined as assistants to physicians despite the increasing complexity and specialization of nursing practice [36–42]. As actual clinical responsibilities expanded, discrepancies between legal definitions and workplace realities generated institutional uncertainty and heightened legal risks within medical settings. Demands for clearer legal recognition and clarification of nursing roles consequently intensified over time.

Concerns regarding healthcare quality and patient safety further strengthened the problem stream. Persistent workforce shortages, frequent resignations, and the growing number of inactive licensed nurses raised concerns regarding continuity and stability in healthcare delivery [115, 116]. These concerns became increasingly linked to broader discussions surrounding staffing standards, working environments, and long‐term sustainability of the healthcare workforce.

Demographic and institutional transformations also contributed to the growing salience of nursing‐related issues. Rapid population aging and the increasing prevalence of chronic diseases accelerated the shift from hospital‐centered treatment toward community‐based and long‐term care systems [22, 43–45]. Supporters of the Nursing Act argued that the existing legal framework was insufficient to accommodate these structural changes and that expanded nursing responsibilities required stronger institutional and legal support.

Within the MSF, policy problems gain agenda status not simply because objective conditions exist, but because particular issues become politically visible through framing, interpretation, indicators, and focusing events [72, 76, 113]. In the case of the Nursing Act, nursing‐related issues gradually shifted from being perceived primarily as occupational grievances to being framed as broader concerns involving public health, patient safety, and healthcare governance. This reframing expanded the political legitimacy of demands for legislative reform.

Focusing events played a particularly important role in intensifying the problem stream. The 2015 MERS outbreak exposed institutional weaknesses in infection control and healthcare workforce management, while the COVID‐19 pandemic significantly amplified public attention toward the role of nurses within crisis response systems [120–131]. During the pandemic, nurses became highly visible frontline actors, and longstanding structural problems—including understaffing, excessive workloads, burnout, and unstable working environments—received heightened social and political attention. These developments strengthened public recognition that existing institutional arrangements were insufficient to support sustainable healthcare delivery during national health emergencies.

Policy feedback and statistical indicators further reinforced recognition of the problem. South Korea continued to exhibit workforce imbalance and relatively vulnerable nursing workforce conditions compared with OECD standards [24, 43, 132]. In addition, high turnover rates and low retention among clinical nurses highlighted structural instability within the nursing workforce [116, 133, 134]. These indicators functioned as important sources of policy feedback supporting claims that institutional and legislative intervention had become necessary.

Taken together, the convergence of focusing events, statistical indicators, workforce instability, and changing healthcare demands substantially strengthened the problem stream surrounding the Nursing Act. In particular, the COVID‐19 pandemic transformed longstanding professional concerns into issues of national policy urgency, thereby contributing to the opening of a policy window for legislative change.

### 4.2. The Policy Stream: Evolution and Competition of Nursing Act Proposals

Policy alternatives related to the Nursing Act evolved through prolonged processes of proposal, revision, negotiation, and conflict across multiple legislative periods. The content and scope of the legislation changed substantially over time in response to shifting healthcare demands, political conditions, and interprofessional disputes [39, 74, 75, 135–139]. Early proposals focused primarily on clarifying nurses’ legal roles and strengthening professional recognition, whereas later versions incorporated broader institutional and workforce‐related provisions.

Initial proposals introduced during the 17th National Assembly included provisions concerning nursing role definitions, institutional support mechanisms, and limited forms of independent nursing practice [14, 140]. During the 20th National Assembly, policy discussions expanded toward establishing a more comprehensive legal framework separate from the Medical Service Act, including workforce support systems and institutional protections for nursing personnel [39, 111, 141].

Legislative debate intensified during the 21st National Assembly as multiple versions of the Nursing Act were proposed, revised, and consolidated [142–145]. Major points of conflict included the interpretation of “assistance in medical treatment,” the legal status of nursing assistants, workforce support measures, and inclusion of the term “community.” Although a consolidated bill ultimately passed the National Assembly, the legislation was vetoed by the president before enactment [59, 141].

The version enacted during the 22nd National Assembly reflected both institutional compromise and strategic adaptation following earlier legislative conflict [146–148]. In particular, the final Act clarified provisions concerning clinical support duties performed under physician supervision and revised several highly contentious expressions that had generated political resistance during previous legislative stages [149–152].

The evolution of Nursing Act proposals illustrates the fluid and competitive nature of the policy stream within the MSF. Policy alternatives were repeatedly modified in response to concerns regarding political feasibility, stakeholder opposition, and changing institutional conditions [72, 76, 153, 154]. Controversial provisions were softened, reinterpreted, or strategically reframed in order to improve legislative acceptability and sustain policy viability within a highly contested political environment.

The policy stream was shaped by interactions among a heterogeneous policy community composed of professional associations, scholars, legislators, bureaucrats, and civil society organizations [7, 74, 103, 135–137, 155–160]. Among these actors, the KNA played the most sustained and influential role by continuously promoting legislative proposals, organizing policy forums, conducting advocacy activities, and mobilizing political support for the Nursing Act [7, 155, 156]. Researchers and policy experts contributed legal and institutional arguments supporting the legislation, while government agencies, including the Ministry of Health and Welfare, frequently acted as mediators during interprofessional disputes [150, 157–165].

Public hearings, policy debates, and coalition activities further expanded the visibility of competing policy alternatives [166–170]. Coalitions supporting the Nursing Act contributed to strengthening the social legitimacy of the legislation and reinforced the argument that nursing‐related issues involved broader questions of healthcare governance and public health rather than narrow occupational interests alone.

One of the most contentious issues within the policy stream concerned whether nursing‐related problems should be addressed through revisions to the existing Medical Service Act or through enactment of a separate Nursing Act [4, 136, 171]. Opponents, particularly the KMA, argued that separate legislation would fragment healthcare governance and intensify interprofessional conflict [46, 48]. Supporters, however, maintained that the physician‐centered structure of the existing Medical Service Act could no longer adequately accommodate the expanding scope and complexity of nursing practice [10, 172].

The term “community” became particularly controversial during debates in the 21st National Assembly [173–179]. Supporters viewed the provision as reflecting the growing importance of community‐based and long‐term care systems, whereas opponents interpreted it as a potential basis for independent nursing practice outside physician supervision. Competing interpretations of the term intensified political conflict and contributed to broader disputes regarding professional boundaries and institutional authority.

Within the MSF, controversy surrounding the “community” clause illustrates how ambiguity within policy proposals may destabilize stream coupling. Failure to reconcile competing interpretations weakened political support for the legislation and contributed to the eventual presidential veto during the 21st National Assembly [14, 59]. By contrast, revisions introduced during the 22nd National Assembly reduced interpretive ambiguity and improved political feasibility, thereby facilitating renewed policy convergence.

Additional controversy emerged regarding PA nurses and the interpretation of the phrase “assistance in medical treatment” under the Medical Service Act [38, 180, 181]. Disputes also arose concerning the educational qualifications and institutional status of nursing assistants [182–184]. These debates reflected broader struggles over professional jurisdiction, authority, and institutional control within the Korean healthcare system.

Overall, the policy stream surrounding the Nursing Act demonstrates how policy alternatives are continuously reshaped through negotiation, conflict, and strategic adaptation. The legislative trajectory of the Nursing Act reflects not only changes in policy content but also broader contestation over healthcare governance, professional authority, and institutional legitimacy in South Korea.

To clarify the evolution and variation of proposals, the following table compares the main provisions contained in several Nursing Act bills submitted across legislative sessions [14, 39, 59, 104, 140–149, 185–188] (Table 2).

**TABLE 2:** Comparison of key provisions in major Nursing Act bills.

Key provisions	2005 Bill (17th National Assembly)	2021 Bill (Early 21st National Assembly)	2023 Bill (21st National Assembly) (presidential veto)	2024 Final Enacted Act (22nd National Assembly)
Definition of nurses’ roles	• Defined the duties and responsibilities of nurses • Attempted independent systematization of nursing through separate legislation	• Stated the purpose and definition of nursing and nurses’ rights	• Emphasized enhancing nursing professionalism and public health promotion	• Emphasized professionalism and public health promotion within a revised framework
Scope of “community” services	• Included some community‐based services (e.g., home visits)	• Differences in inclusion and scope across bills; ongoing controversy	• Explicitly included the term “community” (core issue)	• The term “community” was removed
Assistance in medical treatment	• Focused on “assisting in medical treatment”	• Used broad terms like “tasks necessary for patient care,” causing controversy	• Defined as “assistance in medical treatment under the supervision of physicians, dentists, and oriental medicine doctors,” aligned with the Medical Service Act	• Introduced new clause: “clinical support duties based on a physician’s professional judgment and general supervision and delegation”
Qualification/education for nursing assistants	• No explicit regulations on nursing assistants	• Debated educational standards (e.g., high school diploma) and training institution standards	• Included licensing notification and accreditation of training institutions	• Maintained existing Medical Service Act structure; some issues delegated to subordinate regulations
Improvement of working conditions and support	• Included indirect improvement measures (e.g., policy review committee)	• Called for establishing a Nursing Workforce Support Center and improving conditions	• Included provisions such as dedicated nurse educators and integrated nursing care services	• Specified comprehensive support measures, including workforce training, supply‐demand, working environment improvements, and operation of the Support Center
Physician assistant (PA) nurses	• No explicit mention	• No explicit mention	• No explicit mention	• A new clause on “clinical support duties” provides the legal foundation for institutionalizing the role of PA nurses (clinical support nurses)

### 4.3. Political Stream: The Political Dynamics Surrounding the Nursing Act

The politics stream surrounding the Nursing Act was shaped by changing public sentiment, organized interest‐group conflict, partisan realignment, and shifts within the executive branch [10, 183, 189]. Within the MSF, these political conditions played a decisive role in determining whether legislative momentum could be sustained, delayed, or interrupted.

Public opinion became increasingly favorable toward the Nursing Act during and after the COVID‐19 pandemic. Nurses gained heightened public visibility as frontline healthcare workers, while concerns regarding workforce shortages and long‐term healthcare system sustainability intensified [120–123]. Public support for improving nurses’ working conditions and strengthening legal protections expanded considerably during this period. Survey findings also indicated growing public support for legislative reform related to nursing workforce issues [170, 190]. These developments contributed to the formation of a political environment increasingly receptive to the Nursing Act.

The KNA actively sought to translate favorable public sentiment into political momentum through advocacy campaigns, demonstrations, coalition‐building activities, and media engagement [114, 119, 156, 166–170, 191]. Through these efforts, the KNA attempted to broaden political support for the legislation and frame the Nursing Act as an issue involving healthcare governance, workforce sustainability, and patient safety rather than a narrow professional interest.

At the same time, the KMA emerged as the most influential opposition actor [46, 48, 49, 192]. The KMA argued that the Nursing Act could intensify interprofessional conflict, weaken the physician‐centered healthcare system, and potentially legitimize unauthorized medical practices. Opposition groups engaged in lobbying activities, public campaigns, and collective mobilization in an effort to block the legislation [46, 48, 49, 192–195]. As a result, the political stream surrounding the Nursing Act became increasingly polarized between competing professional groups.

Additional conflict emerged among allied health professions and nursing assistant organizations [189, 196–200]. These groups expressed concerns regarding professional boundaries, workforce qualifications, and possible encroachment upon their institutional roles. Consequently, debate surrounding the Nursing Act expanded into broader disputes concerning professional authority, jurisdiction, and healthcare governance within the Korean healthcare system.

Partisan dynamics also shifted considerably throughout the legislative process. The Democratic Party of Korea consistently supported the Nursing Act and played a central role in advancing the legislation during the 21st and 22nd National Assemblies [201–203]. In contrast, the People Power Party initially demonstrated partial support but later opposed the legislation during the 21st National Assembly and ultimately supported the presidential veto request [59, 184, 204–206]. Political positioning changed again following the 2024 healthcare service vacuum, when the ruling party moved toward compromise and bipartisan negotiation amid growing concerns regarding healthcare system stability [24, 67, 70, 147, 164, 180].

The executive branch exerted particularly strong influence over the political trajectory of the Nursing Act. The Ministry of Health and Welfare frequently acted as a mediator during interprofessional conflict and proposed compromise‐oriented alternatives [141, 157, 159, 164, 207]. The most consequential political intervention occurred when President Yoon Suk‐yeol vetoed the Nursing Act passed during the 21st National Assembly [59–61, 208]. Although the veto temporarily halted legislative progress, it also intensified political attention and mobilization surrounding the issue [209].

Within the MSF, the presidential veto illustrates how executive authority may disrupt stream convergence even after substantial legislative alignment has been achieved. Although the problem and policy streams had converged sufficiently to enable passage through the National Assembly, the executive branch interrupted the policy process through the use of veto power. This suggests that stream coupling may remain unstable within presidential systems characterized by strong executive authority and high levels of political polarization.

Political conditions shifted again during the 2024 healthcare service vacuum caused by the collective resignation of medical residents protesting medical school quota expansion [24, 62–70, 180]. The crisis significantly increased the political salience of nursing workforce issues and altered governmental calculations regarding healthcare system stability. In response, both the ruling party and the executive branch adopted more conciliatory positions toward the Nursing Act, facilitating bipartisan negotiation and eventual enactment.

Electoral cycles and major political events further contributed to fluctuations within the politics stream [210–212]. Presidential elections, legislative turnover, and shifting party strategies repeatedly altered the political feasibility of the legislation. Individual legislators, particularly lawmakers with nursing backgrounds, also functioned as influential policy entrepreneurs by sponsoring bills, organizing hearings, and maintaining legislative momentum across multiple National Assembly sessions [14, 39, 59, 104, 140–148].

Overall, the politics stream surrounding the Nursing Act was characterized by prolonged interprofessional conflict, fluctuating partisan alignment, and significant executive intervention. The eventual enactment of the legislation became possible only after changes in public sentiment, political incentives, and healthcare system pressures generated a more favorable environment for policy convergence.

### 4.4. Coupling of Streams: The Policy Window and the Role of Policy Entrepreneurs

The enactment of the Nursing Act illustrates how policy change occurs when the problem, policy, and politics streams converge during a limited window of opportunity [72, 76, 113, 153]. Although policy windows emerged repeatedly throughout the legislative process, convergence among the streams remained unstable across much of the two‐decade period.

Several focusing events contributed to opening policy windows surrounding the Nursing Act. Earlier events, including the 2015 MERS outbreak, exposed institutional weaknesses within the healthcare system and stimulated discussion regarding workforce stability and nursing roles [128, 129, 213]. However, the COVID‐19 pandemic represented the most significant turning point. The pandemic dramatically increased public visibility of nurses, intensified concern regarding healthcare workforce sustainability, and elevated nursing‐related issues within the national policy agenda [120–127].

Electoral cycles and legislative deadlines also created temporary opportunities for policy advancement [72, 77, 211, 212]. During presidential and legislative elections, nursing‐related issues received increased political attention through campaign pledges and partisan competition. In addition, approaching legislative deadlines within National Assembly sessions generated procedural pressure that accelerated negotiation and policy deliberation [104, 141–145].

The most decisive policy window emerged during the 2024 healthcare service vacuum caused by the collective resignation of medical residents protesting medical school quota expansion [24, 62–70, 180, 214–216]. The resulting strain on healthcare delivery substantially increased institutional recognition of nurses’ roles in maintaining clinical operations and altered political calculations regarding workforce policy. In response, both the government and the ruling party adopted more conciliatory positions toward the Nursing Act, facilitating bipartisan agreement and eventual enactment.

Within the MSF, this episode demonstrates how focusing events may simultaneously strengthen the problem stream and reshape the politics stream [84–87, 217, 218]. The healthcare service vacuum transformed long‐standing nursing workforce concerns into issues of immediate national urgency, thereby enabling stronger alignment among the streams.

At the same time, the legislative trajectory of the Nursing Act demonstrates that stream convergence may remain fragile even after substantial policy advancement. During the 21st National Assembly, the COVID‐19 pandemic strengthened the problem stream and increased public support for legislative reform related to nursing workforce issues [119, 124, 126, 127, 156]. Policy entrepreneurs also succeeded in advancing revised legislative proposals, enabling a level of convergence sufficient for passage through the National Assembly.

However, the subsequent presidential veto disrupted the coupling process despite legislative approval [59–61, 141, 208]. This episode illustrates an important institutional limitation of stream convergence within presidential systems. Although the problem, policy, and legislative political streams had aligned to a considerable degree, executive opposition ultimately interrupted policy adoption through the exercise of veto power. In this respect, the veto functioned as a destabilizing mechanism that weakened stream coupling at the final stage of the policy process.

The role of policy entrepreneurs was central throughout this process. The KNA served as the most persistent policy entrepreneur by continuously promoting legislative reform through advocacy campaigns, coalition‐building, public mobilization, and political lobbying [1, 119, 155, 156]. Through these activities, the KNA consistently linked nursing‐related issues to broader concerns involving healthcare quality, workforce sustainability, and public health governance.

Members of the National Assembly also played important entrepreneurial roles across multiple legislative periods [14, 39, 59, 104, 140–148]. Legislators with nursing backgrounds were particularly influential because they combined professional expertise with political legitimacy, allowing them to sustain legislative momentum despite repeated setbacks [219, 220]. Civil society coalitions supporting the Nursing Act further contributed by mobilizing public support and expanding the social legitimacy of the legislation [114, 166–170, 191].

The eventual enactment of the Nursing Act during the 22nd National Assembly resulted from convergence among strengthened problem recognition, revised policy alternatives, and a substantially altered political environment. The healthcare service vacuum accelerated this convergence by reshaping political incentives and increasing institutional recognition of the nursing workforce’s importance. Policy entrepreneurs successfully leveraged this opportunity at a moment when political resistance had weakened sufficiently to permit enactment.

Overall, the legislative trajectory of the Nursing Act demonstrates that policy windows alone do not guarantee policy adoption. Rather, sustained convergence among streams, together with strategic actions by policy entrepreneurs, appears necessary for legislative success. The case further suggests that strong executive veto power within presidential systems may destabilize stream convergence even after substantial legislative agreement has been achieved.

## 5. Discussion

This study applied Kingdon’s MSF to examine the legislative trajectory and postenactment policy developments of the Nursing Act in South Korea. The findings indicate that the enactment process was shaped by prolonged interaction among the problem, policy, and politics streams, as well as by repeated failures and reconfigurations of stream convergence across multiple legislative periods.

Within the problem stream, longstanding issues, including poor working conditions, workforce instability, ambiguity in nursing roles, and concerns regarding healthcare quality, gradually evolved from occupational grievances into broader public policy concerns [2, 14, 18, 22, 103]. Focusing events such as the MERS outbreak, the COVID‐19 pandemic, and the 2024 healthcare service vacuum substantially increased visibility and political urgency surrounding these issues [120–129]. These crises exposed structural weaknesses within the healthcare system and contributed to reframing nursing‐related problems as matters of healthcare governance and system sustainability rather than profession‐specific concerns alone [221].

Within the policy stream, legislative proposals surrounding the Nursing Act evolved through continuous revision, negotiation, and conflict [7–9, 104, 140–149]. Particularly significant was controversy surrounding the term “community,” which generated competing interpretations regarding professional boundaries and institutional authority. This ambiguity complicated stream convergence by intensifying political resistance and contributing to the presidential veto during the 21st National Assembly [59, 96, 97, 141, 222, 223]. Revisions introduced during the 22nd National Assembly subsequently reduced interpretive ambiguity and improved political feasibility, thereby facilitating renewed convergence between the policy and political streams [149–152].

The politics stream was characterized by persistent conflict among organized interest groups, shifting partisan dynamics, and substantial executive intervention [46, 48, 49, 189, 201–203, 205, 206]. Public opinion became increasingly favorable toward the Nursing Act following the COVID‐19 pandemic, while the KNA mobilized extensive advocacy networks to expand political support for legislative reform [114, 119, 156, 166–170, 191]. At the same time, opposition from physician organizations and related professional groups generated sustained political polarization throughout the legislative process.

One of the most significant findings of this study concerns the role of presidential veto power within the policy process. During the 21st National Assembly, convergence among the problem, policy, and legislative political streams became sufficiently strong to permit passage of the Nursing Act through the National Assembly. Nevertheless, the presidential veto ultimately interrupted policy adoption [59–61, 141]. This finding suggests that, within presidential systems characterized by strong executive authority, stream convergence may remain unstable even after substantial legislative agreement has been achieved [208].

From the perspective of the MSF, the veto may be interpreted as a form of institutional “decoupling” in which executive intervention disrupts otherwise favorable stream alignment. This finding extends existing applications of the MSF by suggesting that institutional characteristics associated with presidential systems may complicate or destabilize policy convergence during later stages of the legislative process. In this respect, the Korean case illustrates how executive authority may function not merely as part of the politics stream, but as a decisive institutional mechanism capable of reshaping policy trajectories even after legislative convergence has occurred.

The findings also demonstrate the critical role of policy entrepreneurs in sustaining legislative momentum during prolonged periods of institutional conflict. The KNA consistently functioned as the most influential policy entrepreneur by reframing nursing‐related issues in broader public health terms, mobilizing coalitions, and strategically leveraging focusing events to strengthen political support [1, 119, 155, 156]. Legislators with nursing backgrounds and civil society coalitions further contributed to stream coupling by maintaining issue visibility and legislative continuity across multiple National Assembly sessions [114, 166–170, 191, 219, 220].

The 2024 healthcare service vacuum ultimately functioned as the decisive policy window that enabled enactment of the Nursing Act [24, 62–70, 180, 214–216]. The crisis altered political incentives by increasing institutional dependence on the nursing workforce and intensifying concerns regarding healthcare system stability. Under these conditions, political resistance weakened sufficiently to allow convergence among the streams.

The case of the Nursing Act provides several broader implications for healthcare governance and workforce policy. First, healthcare workforce policy should not be understood solely as a matter of professional interests, but as an issue directly related to institutional resilience and long‐term sustainability of healthcare systems. Second, prolonged interprofessional conflict may significantly delay policy adaptation even when structural healthcare demands have already changed. Third, the findings suggest that executive veto power within presidential systems may operate as an important institutional variable shaping policy trajectories beyond legislative approval alone.

The study also carries implications for nursing leadership and policy advocacy in aging societies facing workforce shortages and increasing healthcare demands. The Korean case demonstrates that nursing organizations may strengthen policy influence by strategically reframing occupational issues as broader public health and healthcare governance concerns while simultaneously building coalitions extending beyond professional boundaries.

### 5.1. Policy Implications and Recommendations

Based on the findings of this study, several policy implications may be drawn for future healthcare reform processes.

First, institutional mechanisms supporting sustained interprofessional dialogue should be strengthened from the early stages of legislative development. The prolonged conflict surrounding the Nursing Act demonstrates that unresolved disputes regarding professional boundaries and institutional authority may substantially delay policy adaptation even when structural healthcare demands are widely recognized.

Second, policymakers should develop more flexible and responsive governance strategies capable of adapting to sudden focusing events such as public health crises, workforce instability, and disruptions in healthcare delivery systems. The COVID‐19 pandemic and the 2024 healthcare service vacuum illustrate how rapidly changing healthcare conditions may transform policy priorities, political incentives, and legislative feasibility within a relatively short period of time.

Third, healthcare workforce reform should be grounded in systematic empirical evidence regarding staffing conditions, workforce sustainability, and healthcare delivery outcomes. Evidence‐based policy communication may help reduce political polarization and strengthen public legitimacy during highly contested reform processes involving multiple professional groups and institutional stakeholders.

Fourth, greater institutional attention should be directed toward postlegislative governance and implementation conflict. Ongoing disputes surrounding the implementation of clinical support duties following enactment of the Nursing Act demonstrate that legislative success does not necessarily resolve policy conflict, but often initiates a new phase of negotiation regarding implementation, authority, professional jurisdiction, and institutional control.

Finally, the findings suggest broader implications for healthcare governance in aging societies facing increasing workforce shortages and rising healthcare demand. The Korean case indicates that successful healthcare workforce reform requires not only legislative change itself, but also sustained coordination among professional groups, political institutions, and healthcare governance systems capable of responding to evolving social and demographic conditions.

### 5.2. Directions for Future Research

Several directions for future research emerge from this study.

First, additional research is needed to further examine how institutional characteristics of presidential systems influence stream convergence and policy stability within the MSF. Comparative studies involving parliamentary and presidential systems may help clarify how executive authority shapes policy outcomes during later stages of the legislative process.

Second, future studies should examine the implementation phase of the Nursing Act and assess its long‐term effects on nursing practice, workforce conditions, healthcare governance, and interprofessional relations. Such research would help identify the extent to which legislative reform produces substantive institutional change in practice.

Third, comparative research involving other healthcare professions and international healthcare systems could provide broader insight into how professional conflict, workforce governance, and policy entrepreneurship influence healthcare reform processes across different institutional contexts.

## Author Contributions

Donguk Lee conceptualized the study, collected and analyzed the data, and wrote the manuscript.

## Funding

This research received no external funding.

## Ethics Statement

This study did not involve human participants, animals, or identifiable human data; therefore, ethical approval was not required.

## Conflicts of Interest

The author declares no conflicts of interest.

## Data Availability

Data sharing is not applicable to this article as no datasets were generated or analyzed during the current study.
